# Automated Crystal Structure Determination Has its Pitfalls: Correction to the Crystal Structures of Iodine Azide

**DOI:** 10.1002/anie.202105666

**Published:** 2021-06-29

**Authors:** Ulrich Müller, Sergei Ivlev, Stephan Schulz, Christoph Wölper

**Affiliations:** ^1^ Fachbereich Chemie Philipps-Universität 35032 Marburg Germany; ^2^ Institute of Inorganic Chemistry and Center for Nanointegration Duisburg–Essen (CENIDE) University of Duisburg–Essen Universitätsstraße 5–7 45141 Essen Germany

**Keywords:** crystal structures, feigned misorder, incorrect lattice parameters, iodine azide

## Abstract

Previously published crystal structure determinations of two modifications of iodine azide (IN_3_) are corrected. In the original determinations, the very weak X‐ray reflections with odd k Miller indices had been discarded, resulting in too small unit cells and models with misordered, partly occupied atomic positions. Using the original diffraction data, refinements with the correct unit cells yield structures of polymeric (−I−N_3_−)_n_ chains that are interlocked to layers. A skilled look at the primary X‐ray data is always recommended to overcome the lack of crystallographic expertise of computers at automated structure determinations.

## Graphical abstract

In the previous crystal structure determinations of both modifications of iodine azide half of the X‐ray reflections were missed, resulting in incorrect unit cells, incorrect space groups, and unsatisfactory structural models. Using the old data, but including the missed weak reflections, sensible structures were determined. It pays off to take a skilled look at the primary X‐ray data and not to rely solely on computer generated models.

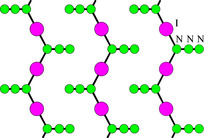

Halogen azides XN_3_ (X=F, Cl, Br, I) have been known for decades. They are explosive compounds with a remarkable chemical reactivity.^[1]^ They were extensively characterized by different spectroscopic methods^[2]^ and by single‐crystal X‐ray diffraction (ClN_3_,^[2]^ BrN_3_,^[3]^ and IN_3_[^2^, ^4^]). ClN_3_ forms a chain‐like polymer in the solid state, BrN_3_ a helical structure due to formation of intermolecular Br⋅⋅⋅N_α_ and N_β_⋅⋅⋅N_γ_ interactions, while two modifications were reported for IN_3_. Its crystal structure was first determined by X‐ray diffraction in 1993;^[4]^ we call it α‐IN_3_. In following crystal structure determinations this structure was confirmed, and a second modification was found that we call β‐IN_3_.^[2]^ Crystals of both polymorphs were obtained by slow sublimation of cooled (0 °C) samples of IN_3_ onto a cooled finger (−10 °C),^[5]^ α‐IN_3_ also from 4 °C to 0 °C.^[4]^ Both modifications were reported to crystallize in the space group *Pbam*, and in both cases a misorder^[6]^ has been described, with half‐occupied nitrogen positions residing on mirror planes, and with azido groups whose terminal atoms N3 and N3A collide with one another (Figure 1). That is an unsatisfactory structural model.

**Figure 1:**
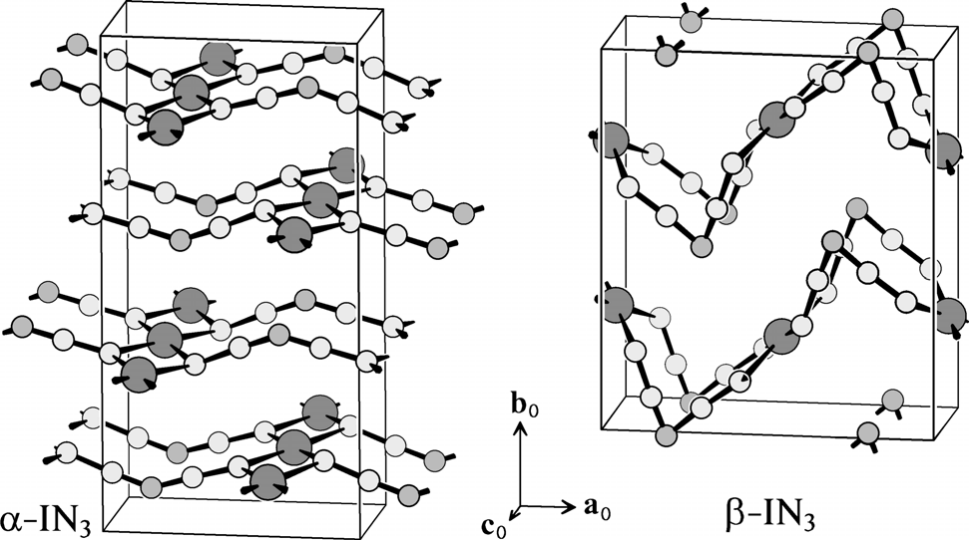
Previously reported unit cells with the alleged misordered (incorrect) structures of IN_3_. N atoms in light gray have occupancies of 1/2 . N atoms are at *z*_0_=0, iodine atoms at *z*_0_=1/2 .^[7]^.

Reasonable structures of both modifications, α‐IN_3_ and β‐IN_3_, can be derived from the published structures by doubling of the lattice parameters *c*_0_. However, then in all crystal structure determinations hitherto half of the X‐ray reflections *h*_0_ *k*_0_ *l*_0_ must have been missed, namely all those with odd indices *l*_0_ referring to the doubled unit cells. In α‐IN_3_ and β‐IN_3_ all nitrogen atoms are situated on mirror planes with the iodine atoms exactly halfway in between. This causes all reflections with odd *l* indices to be very weak and only discernible at lower diffraction angles. A renewed close inspection of the X‐ray diffraction patterns in the form of simulated precession patterns extracted from the original X‐ray diffraction files^[2]^ indeed showed the presence of very weak reflections observable only at lower diffraction angles (Figure 2). Additionally, some spurious reflections were observed due to differently aligned attached crystal fragments. Their existence cannot be avoided taking into account that iodine azide is extremely difficult and dangerous to handle, and that this had to be done at low temperatures. In such a case the software of the diffractometer^[8]^ may have difficulties in finding the correct unit cell in presence of the spurious as well as the very weak reflections. That happened in the previous attempts of structure determination and entailed the determination of the wrong unit cells and wrong space groups. We now redetermined the unit cells and performed new structure refinements, taking into account the weak reflections.[^8^, ^9^]

**Figure 2:**
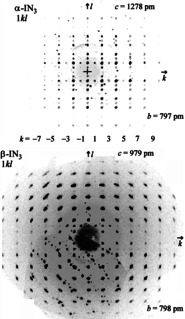
Simulated precession patterns of the reciprocal planes 1*kl* of the IN_3_ modifications, extracted from the measured data. There are only a few weak reflections for odd *k* values, and there are spurious reflections form attached crystal fragments. Compared to the original data, *k* and *l* are interchanged.

Although it is possible to directly transform the wrong smaller unit cell into the correct one by doubling the corresponding lattice parameter, we also tested the automatic algorithms to find out whether adjusting the parameters may lead to the correct results. In fact, the correct unit cells can be automatically found using a lower *I*/sigma threshold value for peak search and using a sufficient number of peaks (we tested peak harvesting from 150 frames: 50 frames in each of the first three runs). A threshold of 6 was enough, but a too low value led again to wrong unit cells due to the spurious reflections. No other special treatment of the datasets was required.

The true space groups are *Pnam*, which are subgroups of *Pbam* with doubled *c*.^[10]^ We transformed this to the conventional setting *Pnma* by exchanging *b* and *c*, so that the doubled lattice parameter is now *b* and the weak reflections are now those with odd *k* indices, resulting in new lattice parameters at 100 K for α‐IN_3_ (*a=*655.56(3) pm, *b=*796.88(4) pm, *c=*1277.90(5) pm) and β‐IN_3_ (*a=*845.95(6) pm, *b=*798.22(5) pm, *c=*978.86(7) pm), respectively. More crystallographic details are given in the Supporting Information.

Both α‐IN_3_ and β‐IN_3_ consist of polymeric chain molecules, having the shape of a double comb with teeth (azide groups) pointing to opposite sides. The teeth of neighbouring molecules are interlocked, resulting in corrugated layers perpendicular to **c** (in the new setting). The corrugation is much more pronounced in β‐IN_3_ (Figure 3).

**Figure 3:**
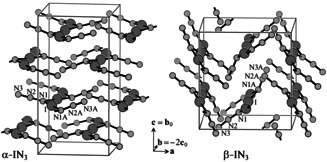
Correct unit cells of the IN_3_ modifications. N atoms are at *y*=1/4 and *y*=3/4 , iodine atoms at *y*=0 and *y*=1/2 .^[7]^

Every iodine atom has a linear coordination with two azido groups. This is in accordance with the expectations of the electron pair repulsion theory when we assume three lone electron pairs at each iodine atom. The I−N bond lengths are in the range 227.1±0.4 pm. In NI_3_⋅NH_3_, which also contains the same kind of polymeric (−I−N−)_*n*_ strands with linearly coordinated iodine atoms, it is 230 pm.^[11]^ The zigzag chains of the iodine atoms with the N1 and N1A atoms are almost exactly planar. The azido groups are nearly linear, the bond angles at the central N atoms are summarized in Table 1.

**Table 1:** Selected bond lengths /pm and angles /° of both IN_3_ polymorphs.

	I−N	N1−N2	N1A−N2A	N1−N2−N3	N1A−N2A−N3A
α‐IN_3_	227.1(3), 227.5(2)	121.7(9)	123.6(9)	179.8(8)	177.4(8)
β‐IN_3_	227.5(2), 226.9(2)	121.0(6)	123.7(5)	179.6(5)	177.4(5)

The bond lengths in the azido groups are N1−N2 122.5±1.5 pm and N2−N3 113.3±0.7 pm and correspond to those in covalently bonded azido groups.^[12]^

The atoms N1 and N1A and the three atoms bonded to each of them are not exactly coplanar. Relative to the plains I−N1−I′ and I−N1A−I′, respectively, the azido groups are inclined by the values given in Table 2.

**Table 2:** Inclination angles of the azido groups relative to the I−αN−I′ planes.

		N1−N2	N1A−N2A	
	α‐IN_3_	10(1)°	29(1)°	
	β‐IN_3_	9(1)°	29(1)°

The crystals probably consist of multiple antiphase domains that are stacked in the direction of **c** and that differ by having the layers shifted by 1/2 **b**. But, contrary to twinning, antiphase domains cannot be detected by X‐ray diffraction unless their thickness in the **c** direction is very small. In that case diffuse streaks parallel to c* would be observed at odd *k* indices instead of the weak Bragg reflections. We did not detect any diffuse streaks.

The computer programs that assist the X‐ray data collection and the crystal structure determination have become powerful tools. It has become a widespread practice to let the programs do most of work with the aid of default settings. However, the lack of crystallographic expertise of computers is also a source of pitfalls that cause the emergence of errors. Some structures exhibit “superstructure reflections” in their X‐ray diffraction diagrams, that is, rather weak reflections in between the main reflections. If these weak reflections are missed or sorted out by the computer, an incorrect unit cell and an incorrect space group result, and the following structural model is incorrect in spite of an excellent agreement between observed and calculated structure factors. It is an alarm signal if the deduced structural model exhibits a feigned misorder or other suspicious features. The true space group then is a subgroup of the incorrectly assumed space group.^[13]^ It always pays off to take a skilled look at the primary X‐ray data. This case also nicely stresses the importance of the availability of primary X‐ray data. A regular *hkl* file would not have been sufficient to improve the published structure model.

## Conflict of interest

The authors declare no conflict of interest.

## Supporting information

As a service to our authors and readers, this journal provides supporting information supplied by the authors. Such materials are peer reviewed and may be re‐organized for online delivery, but are not copy‐edited or typeset. Technical support issues arising from supporting information (other than missing files) should be addressed to the authors.

Supporting Information
