# Supplementary material for: Automated Crystal Structure Determination Has its Pitfalls: Correction to the Crystal Structures of Iodine Azide
Source: Angew Chem Int Ed Engl. 2021 Jun 29;60(32):17452–4. doi: 10.1002/anie.202105666 (PMC8361933; doi:10.1002/anie.202105666)
Supplement: Supplementary file 1 — Supporting Information [file ANIE-60-17452-s001.pdf]

Angewandte Chemie
Eine Zeitschrift der Gesellschaft Deutscher Chemiker

Supporting Information

Automated Crystal Structure Determination Has its Pitfalls: Correction to the Crystal Structures of Iodine Azide

Ulrich Müller, Sergei Ivlev, Stephan Schulz,* and Christoph Wölper*

anie_202105666_sm_miscellaneous_information.pdf

For details on the preparation and X-ray measurements see [2]. Extraction of the diffraction intensities and unit cell determinations were performed with the diffractometer software,[8] the refinement with SHELXL.[9] Deposition numbers 2080096 (α-IN_3) and 2080097 (β-IN_3) contain supplementary crystallographic data for this paper. These data are provided free of charge by the joint Cambridge Crystallographic Data Centre Fachinformationszentrum Karlsruhe Access Structures service www.ccdc.cam.ac.uk/structures.

Crystallographic Data for iodine azide and details of the structure determinations

	α-IN_3	β-IN_3
Molar mass /g mol^{-1}	168.93	168.93
Space group	*Pnma*	*Pnma*
a /pm	655.56(3)	845.95(6)
b /pm	796.88(4)	798.22(5)
c /pm	1277.90(5)	978.86(7)
V /pm^3	667.6×10^6	661.0×10^6
Z	8	8
Pearson symbol	*oP*32	*oP*32
ρ_{calc} /g cm^{-3}	3.362	3.395
μ /mm^{-1}	9.33	9.43
T /K	100(2)	100(2)
λ /pm	71.07	71.07
Number of reflections	16385	14891
Number of unique reflections	1060	1087
Number of observed unique reflections $I \geq 2\sigma(I)$	704	778
R_{int}, R_σ	0.031, 0.016	0.033, 0.016
Number of parameters	46	46
S, all data	1.13	1.07
$R(F), I \geq 2\sigma(I)$, all data	0.025	0.019
$wR(F), I \geq 2\sigma(I)$, all data	0.075	0.055
$\Delta\rho_{max}$, $\Delta\rho_{min}$ /eÅ^{-3}	1.8, −1.0	1.0, −0.8

Atomic coordinates for α-IN_3

Atom	Position	x	y	z	U_{iso} /pm^2
I	8*d*	0.19551(4)	0.50095(4)	0.35432(2)	144(1)
N1	4*c*	0.0318(10)	$\frac{1}{4}$	0.3610(6)	254(15)
N2	4*c*	−0.1480(9)	$\frac{1}{4}$	0.3846(5)	153(10)
N3	4*c*	−0.3166(10)	$\frac{1}{4}$	0.4064(6)	257(16)
N1A	4*c*	0.3617(10)	$\frac{3}{4}$	0.3436(5)	188(12)
N2A	4*c*	0.5362(9)	$\frac{3}{4}$	0.3802(5)	164(11)
N3A	4*c*	0.6900(10)	$\frac{3}{4}$	0.4099(6)	243(15)

Anisotropic thermal displacement parameters for α-IN_3 at 100 K

Atom	U_{11}/pm^2	U_{22}/pm^2	U_{33}/pm^2	U_{23}/pm^2	U_{13}/pm^2	U_{12}/pm^2
I	143(2)	86(2)	203(2)	−1(1)	−3(1)	14(1)
N1	173(29)	76(30)	51(46)	0	20(27)	0
N2	191(27)	75(30)	191(27)	0	−6(24)	0
N3	209(35)	219(39)	342(43)	0	36(27)	0
N1A	181(28)	79(29)	304(33)	0	−7(24)	0
N2A	198(29)	70(27)	224(28)	0	20(23)	0
N3A	203(34)	262(41)	263(38)	0	4(24)	0

Atomic coordinates for β-IN_3

Atom	Position	x	y	z	U_{iso}/pm^2
I	8*d*	0.50698(3)	−0.00135(2)	0.21588(2)	143(1)
N1	4*c*	0.4174(6)	$\frac{1}{4}$	0.1383(5)	272(10)
N2	4*c*	0.3019(5)	$\frac{1}{4}$	0.0654(4)	173(7)
N3	4*c*	0.1937(6)	$\frac{1}{4}$	−0.0038(6)	2337(9)
N1A	4*c*	0.6035(5)	$\frac{3}{4}$	0.2911(5)	197(8)
N2A	4*c*	0.6506(5)	$\frac{3}{4}$	0.4107(4)	163(7)
N3A	4*c*	0.6987(6)	$\frac{3}{4}$	0.5166(4)	248(10)

Anisotropic thermal displacement parameters for β-IN_3 at 100 K

Atom	U_{11}/pm^2	U_{22}/pm^2	U_{33}/pm^2	U_{23}/pm^2	U_{13}/pm^2	U_{12}/pm^2
I	162(1)	100(1)	168(1)	−10(1)	14(1)	−7(1)
N1	305(24)	134(18)	377(24)	0	−134(21)	0
N2	243(19)	90(16)	186(18)	0	56(16)	0
N3	203(22)	252(24)	243(22)	0	−52(17)	0
N1A	260(22)	124(17)	207(19)	0	−30(16)	0
N2A	153(17)	116(16)	220(19)	0	46(15)	0
N3A	279(25)	206(22)	259(22)	0	−23(18)	0

Bond lengths/pm for iodine azide at 100 K

Bond	α-IN_3	β-IN_3
I–N1	227.1(3)	227.5(2)
I–N1A	226.8(3)	226.9(2)
N1–N2	121.7(9)	121.0(6)
N2–N3	114.0(9)	113.8(6)
N1A–N2A	123.6(9)	123.7(5)
N2A–N3A	113.3(9)	113.6(6)

Bond angles/° for iodine azide at 100 K

Angle	α-IN_3	β-IN_3
N1–I–N1A	178.6(2)	178.3(2)
I–N1–I′	123.4(2)	123.7(2)
I–N1A–I′	122.1(3)	122.1(2)
I–N1–N2	117.9(2)	117.8(1)
I′–N1–N2	117.9(2)	117.8(1)
I–N1A–N2A	115.0(3)	115.0(1)
I′–N1A–N2A	115.0(3)	115.0(1)
N1–N2–N3	179.8(8)	179.6(5)
N1A–N2A–N3A	177.4(8)	177.4(5)

Equivalent position of I′: $x, \frac{1}{2} - y, z$

Group–subgroup relations between the misordered structures and the true structures of iodine azide

The previous structure determinations that had missed the weak reflections require a doubling of the lattice parameters c_0 of the published structures in the space group *Pbam*. To conform with the conventions of the true space groups, we first transform the published structural data from the setting *Pbam* and basis vectors $\mathbf{a}_0$, $\mathbf{b}_0$, $\mathbf{c}_0$ to *Pcma* by exchanging the basis vectors from $\mathbf{a}_0\mathbf{b}_0\mathbf{c}_0$ to $\mathbf{a}_0\overline{\mathbf{c}}_0\mathbf{b}_0$. The false space group *Pbam* then becomes *Pcma*. The true space groups must be subgroups of *Pcma* with doubled basis vector $\mathbf{b} = 2\overline{\mathbf{c}}_0$. There are six such subgroups to be considered, namely *Pcma*, *Pnma* and *Pnmn*, with and without an origin shift of $\frac{1}{4}\mathbf{b}$ as listed at the coordinate transformations in *International Tables A*1.[10] Starting from the original atomic coordinates,[2] we tried refinements in all six subgroups. Only one of them, namely *Pnma* (No. 62) can be satisfactorily refined. The corresponding group–subgroup relations are given in the following Bärnighausen trees.[13] All positions of the nitrogen atoms having half occupancy split into two positions, of which one is fully occupied and the other (at $y - \frac{1}{2}$) remains vacant (not mentioned in the tree). The atomic coordinates given in the boxes are the refined values.

$P2_1/c\,2/m\,2_1/a$

α-IN_3 misordered

I:4*g*	$\frac{1}{2}$N1:4*h*	$\frac{1}{2}$N2:4*h*	$\frac{1}{2}$N1A:4*h*	$\frac{1}{2}$N2A:4*h*	N3:4*h*
.*m*.	.*m*.	.*m*.	.*m*.	.*m*.	.*m*.
0.19546	0.0309	−0.1489	0.3620	0.5372	−0.3092
1	$\frac{1}{2}$	$\frac{1}{2}$	$\frac{1}{2}$	$\frac{1}{2}$	$\frac{1}{2}$
0.35431	0.3603	0.3847	0.3429	0.3800	0.4082

k2

a, 2**b**, **c**

$x, \frac{1}{2}y, z; +(0, \frac{1}{2}, 0)$

$P2_1/n\,2_1/m\,2_1/a$

α-IN_3 correct structure

I:8*d*	N1:4*c*	N2:4*c*	N1A:4*c*	N2A:4*c*	N3:4*c*	N3A:4*c*
1	.*m*.	.*m*.	.*m*.	.*m*.	.*m*.	.*m*.
0.19551	0.0318	−0.1480	0.3167	0.5362	−0.3166	0.6990
0.50095	$\frac{1}{4}$	$\frac{1}{4}$	$\frac{3}{4}$	$\frac{3}{4}$	$\frac{1}{4}$	$\frac{3}{4}$
0.35432	0.3610	0.3846	0.3436	0.3802	0.4064	0.4099

$a = 655.6$ pm, $b = 796.9$ pm, $c = 1277.9$ pm

$P2_1/c\,2/m\,2_1/a$

β-IN_3 misordered

I:4*g*	$\frac{1}{2}$N1:4*h*	$\frac{1}{2}$N2:4*h*	$\frac{1}{2}$N1A:4*h*	$\frac{1}{2}$N2A:4*h*	N3:4*h*
.*m*.	.*m*.	.*m*.	.*m*.	.*m*.	.*m*.
0.50728	0.4177	0.3020	0.6036	0.6512	0.1963
0	$\frac{1}{2}$	$\frac{1}{2}$	$\frac{1}{2}$	$\frac{1}{2}$	$\frac{1}{2}$
0.21579	0.1384	0.0655	0.2906	0.4109	−0.0118

k2

a, 2**b**, **c**

$x, \frac{1}{2}y, z; +(0, \frac{1}{2}, 0)$

$P2_1/n\,2_1/m\,2_1/a$

β-IN_3 correct structure

I:8*d*	N1:4*c*	N2:4*c*	N1A:4*c*	N2A:4*c*	N3:4*c*	N3A:4*c*
1	.*m*.	.*m*.	.*m*.	.*m*.	.*m*.	.*m*.
0.50698	0.4174	0.3019	0.6035	0.6506	0.1938	0.6987
−0.00135	$\frac{1}{4}$	$\frac{1}{4}$	$\frac{3}{4}$	$\frac{3}{4}$	$\frac{1}{4}$	$\frac{3}{4}$
0.21588	0.1383	0.0654	0.2911	0.4107	−0.0381	0.5166

$a = 846.0$ pm, $b = 798.2$ pm, $c = 978.9$ pm

References (Same numbering as in main text)

[2] B. Lyhs, D. Bläser, C. Wölper, S. Schulz, G. Jansen, *Angew. Chem.* **2012**, *124*, 13031–13035; *Angew. Chem. Int. Ed.* **2012**, *51*, 12859–12863.

[8] *APEX*3, Bruker AXS Inc., Madison, Wisconsin, USA, **2018**. R. H. Blessing, *Acta Crystallogr.* A **1995**, *51*, 33–38.

[9] G. M. Sheldrick, SHELXL, *Acta Crystallogr.* C **2015**, *71*, 3–8.

[10] *International Tables for Crystallography* Vol. *A*1, 2nd edition, **2010**, (Eds. H. Wondratschek, U. Müller), Wiley, Chichester, UK.

[13] U. Müller, *Symmetry Relationships between Crystal Structures*, Chapter 17, Oxford Univ. Press, **2013**; *Symmetriebeziehungen zwischen verwandten Kristallstrukturen*, Kapitel 17, Vieweg+Teubner, Wiesbaden, Germany, **2012**; *Relaciones de simetría entre estructuras cristalinas*, Capítulo 17, Editorial Síntesis, Madrid, Spain, **2013**.
